# RNA Viruses, Pandemics and Anticipatory Preparedness

**DOI:** 10.3390/v14102176

**Published:** 2022-09-30

**Authors:** Mariano A. Garcia-Blanco, Eng Eong Ooi, October M. Sessions

**Affiliations:** 1 Department of Biochemistry and Molecular Biology, University of Texas Medical Branch, Galveston, TX 77555, USA; 2 Department of Internal Medicine, University of Texas Medical Branch, Galveston, TX 77555, USA; 3 Institute for Human Infections and Immunity, University of Texas Medical Branch, Galveston, TX 77555, USA; 4 Programme in Emerging Infectious Diseases, Duke-NUS Medical School, Singapore 169857, Singapore; 5 Saw Swee Hock School of Public Health, National University of Singapore and National University Health System, Singapore 117549, Singapore; 6 Viral Research and Experimental Medicine Center, SingHealth Duke-NUS Academic Medical Center, Singapore 169857, Singapore; 7 Department of Pharmacy, National University of Singapore, Singapore 117559, Singapore

**Keywords:** RNA viruses, pandemics, anticipatory preparedness

## Abstract

RNA viruses are likely to cause future pandemics and therefore we must create and organize a deep knowledge of these viruses to prevent and manage this risk. Assuming prevention will fail, at least once, we must be prepared to manage a future pandemic using all resources available. We emphasize the importance of having safe vaccine candidates and safe broad-spectrum antivirals ready for rapid clinical translation. Additionally, we must have similar tools to be ready for outbreaks of RNA viruses among animals and plants. Finally, similar coordination should be accomplished for other pathogens with pandemic potential.

## 1. Introduction

COVID-19 is teaching us valuable lessons regarding pandemics, but it should be clear this does not necessarily imply that we are learning these lessons. We have been taught that, as with Atlantic hurricanes in August, come they must. Pandemics will be with us as long as there are humans (In this essay we focus on human disease, but similar lessons can be applied to infections of other animals and plants). We should have learned that many will emerge from zoonotic pathogens and that we should therefore be mindful and informed about animal health and the interface between humans and animals Regardless of the situation leading to the spill over of SARS-CoV-2 into the general population of Wuhan, it is very likely that very close ancestors of this virus infected bats and possibly other intermediate species [1]. Although the general wisdom is that pandemics do not cause as much devastation as more chronic conditions, such as obesity, cancers or endemic-communicable diseases (e.g., malaria), our present experience should make it self-evident that pandemics can lead to acute and profound crises of health systems, which can increase the morbidity and mortality from many other causes [2,3]. Equally, pandemics can lead to socio-economic catastrophes that enhance inequality, stress social compacts and compound the toll of poverty [4]. As scientists, we may have to seriously evaluate our own roles in pandemics [5]. In this review, we focus on a very specific and important lesson highlighted by COVID-19: most pandemics affecting the world in the last ~100 years have been caused by RNA viruses Although we emphasize RNA viruses here, we note that the recent monkeypox outbreak reminds us about the danger posed by DNA viruses and how similar principles apply to these pathogens [6].

**Figure 1:**
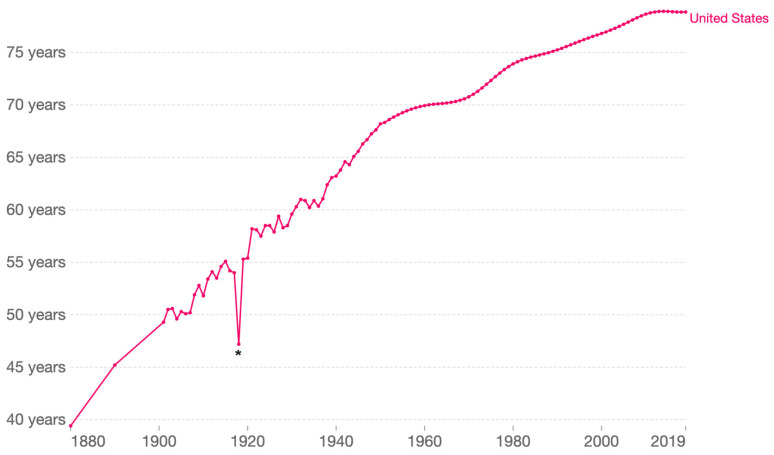
Life expectancy in the United States 1880–2019. Shown is period life expectancy. Note the impact of the 1918 influenza pandemic (*). Taken from OurWorldInData.org/life-expectancy.

RNA viruses are remarkable for their ability to adapt, which makes them capable of exceedingly successful transmission and defense evasion [7]. A few examples starting in the early 20th Century are mentioned here. A member of the *Orthomyxoviridae* family, which are segmented negative-sense single stranded RNA viruses, caused the great influenza pandemic of 1917–1919, usually referred as the 1918 flu or, misleadingly, Spanish flu. This influenza virus dramatically altered human life expectancy around the globe (Figure 1) [8]. A few decades later, in part because of the massive mobilization of human populations and the dissemination of the mosquito vector, *Aedes aegypti*, during World War II, and aided by global warming, the four dengue viruses, positive-sense single stranded RNA viruses of the family *Flaviviridae*, have spread across tropical and temperate regions of the world [9]. The poliomyelitis pandemic, which peaked in the early 1950s before the introduction of the Salk polio vaccine (1955) and then the Sabin vaccine (1963), whose broad adoption resulted in a rapid decline of cases [10]. In the last third of the 20th century, the human immunodeficiency viruses (HIV-1 and HIV-2), lentiviruses of the *Retroviridae* family, caused worldwide devastation [11]. In many countries in Sub-Saharan Africa, the resulting acquired immunodeficiency syndrome (AIDS) led to unprecedented changes in the population demographics and a colossal increase in the number of orphans [12].

It did not take long for an RNA virus to wreak global havoc on the 21st century. Babies from mothers who were infected with the Zika virus—a flavivirus—during pregnancy, were born with congenital Zika syndrome which resulted in physical deformities and childhood developmental delays. A strain of the chikungunya virus—an alphavirus—with a mutation that enabled it to be transmitted efficiently by both *Aedes aegypti* and *Aedes albopictus* emerged from East Africa, and spread eastwards globally to cause acute febrile illness and debilitating joint pains that last for months. Coronavirus disease 2019 (COVID-19), caused by the severe acute respiratory syndrome coronavirus 2, SARS-CoV-2, a member of the *Coronaviridae* family of RNA viruses, significantly altered mortality in many countries in 2020 and 2021, and continues unabated in 2022, causing massive morbidity and mortality [13].

The list above is not meant to be comprehensive and indeed does not mention the many times that RNA viruses have caused large epidemics with terrifying pandemic potential. A salient example of such a threat was the West African Ebola virus epidemic of 2014–2016 caused by the Ebola virus, a member of the *Filoviridae* family of RNA viruses [14]. The lessons provided by these full-blown pandemics and near-misses caused by RNA viruses need to become part of our long-term memory and knowledge. Otherwise, as the American–Spanish philosopher Jorge Agustin (George) Santayana warned in 1905, we will be condemned to repeat the past [15].

Our very recent past can be taken as an example. While the response to COVID-19 involved the very rapid development and deployment of many effective vaccines and therapeutics, it is sad to note that more than 6.5 million have died from this respiratory disease in this pandemic worldwide, and the death toll keeps climbing [13]; the impact of COVID-19 on death rates is likely greater if excess mortality from an over-stressed healthcare infrastructure is also considered [16]. The COVID-19 pandemic has thus negatively impacted life expectancy in many countries, and will likely provide a second significant distortion similar to that shown in Figure 1 for the 1918 influenza pandemic [17]. It is also sobering to imagine what would have happened had the pandemic agent been equally transmissible but possessed the case fatality rate of MERS-CoV [18]. We must do better next time, and to do so we must look at the COVID-19 pandemic and ask how we could have anticipated better.

True anticipation involves preparations at every level of society and the coordination between international entities, governments, industry, and others. While the coordination of disparate disciplines is essential, here we focus on one very specific aspect of preparedness: the high likelihood that a future pandemic will be caused by a pathogenic RNA virus. It is with this focus that we present our thoughts on pandemic anticipation.

## 2. Anticipation

It is impossible to know with complete certainty what the future will bring, so how do we foresee a response to an RNA virus we have never encountered before? The encounters with highly pathogenic betacoronaviruses in the last twenty years can provide us with a guide. From late 2002 to the first half of 2003, SARS-CoV-1 caused an outbreak of severe acute respiratory illness, now known as SARS, often leading to pneumonia and acute respiratory distress syndrome (ARDS)-like findings. This outbreak affected 29 countries and territories and sickened more than 8000, with a case fatality rate of approximately 10% [19]. Starting in 2012, a second betacoronavirus was shown to cause Middle East respiratory syndrome (MERS), which has afflicted more than 2500 in more than 25 countries, and has a case-fatality rate that exceeds 35% [18]. In late 2019, SARS-CoV-2 erupted and eventually engulfed the globe in a disastrous pandemic. SARS-CoV-1, MERS-CoV and SARS-CoV-2 were new viruses, but what we knew about the first two helped prepare us for SARS-CoV-2 [20]. In this essay we posit that there is much more we should have known about SARS-CoV-1 and MERS-CoV and the syndromes they cause, and this additional knowledge could have significantly reduced the suffering and loss of life caused by COVID-19.

SARS-CoV-1 and MERS-CoV are genetic relatives of SARS-CoV-2, and inhabit a neighborhood of viruses that like many neighborhoods, can inform on the behavior of individuals within it [21]. We will develop this concept here, cautious that such a definition of a neighborhood must be driven by facts and not prejudices. For the COVID-19 pandemic, the relevant neighborhood was inhabited by SARS-CoV-1 and MERS-CoV [21]. The neighborhood was also inhabited by distantly related viruses with functional similarities created by convergent evolution, such as influenza A virus (IAV) and respiratory syncytial virus (RSV), which also cause respiratory illnesses [22]. What information from this neighborhood could have been used to prevent the spread of SARS-CoV-2 or to treat those afflicted with SARS-2? This information can be defined as the atlas of knowledge required for anticipatory preparedness.

What are the viral neighborhoods of greatest concern? The answer is not straightforward and must include a consideration that past is prologue. If we did not know about the devastation caused by the ongoing AIDS pandemic, we may not consider lentiviruses among potential culprits, but we must. In the general neighborhood of HIV-1 and HIV-2, we have to also include genetically distinct viruses also capable of sexual and parenteral transmission [23]. Respiratory viruses, which belong to many different families, including the aforementioned coronaviruses, and influenza A viruses, are in almost everyone’s list of potential pandemic culprits [1,24]. Additionally, arboviruses, particularly those transmitted by *Aedes aegypti* and *Aedes albopictus* (e.g., dengue viruses) that are well-adapted to the urban environment, infect a staggering number of humans every year and are continuously on the verge of becoming pandemic [25]. The explosive circumnavigation of the globe by the Chikungunya virus in 2005–2006 and the Zika virus in 2015–2016, provide two remarkable examples of the capacity of arboviruses to ignite pandemics [26,27].

Past is prologue, however, is not good enough, and thus we must add other viruses to extend the neighborhoods of pandemic concern. We should prioritize viruses with high case fatalities, such as hantaviruses which cause respiratory illness, and where there is suspicion about potential human-to-human transmission (e.g., the Andes virus) [28]. Other highly lethal viruses, such as the Ebola virus, have created large epidemics that, although geographically localized, have nonetheless seeded cases in distant countries, raising the specter of a pandemic which could have unimaginable consequences [29].

As a first step, we must prepare a comprehensive list of the concerning viral neighborhoods (we really cannot predict the existence of one specific species) and the virus residents in them, and for each we must develop an atlas of information required for anticipatory preparedness. This list will need to be informed by surveillance for emerging zoonotic pathogens, particularly in those areas of the world that have experienced frequent zoonotic disease outbreaks [30,31,32]. PREDICT, a project of USAID’s emerging pandemic threats program, is one such pre-emptive surveillance system that can provide epidemiological data to shape our proposed list of concerning viral neighborhoods [33]. This information will help us prevent a future pandemic, and if that fails, which is likely, will help us manage it.

## 3. Preventing the Next Pandemic

Since many pandemics are caused by zoonotic pathogens, a prevention barrier is necessary to minimize the chances of the first introduction of a virus to human populations. The sequential appearance of novel coronaviruses provides a good example. Both SARS-CoV-1 and MERS-CoV likely evolved from bat betacoronaviruses which acquired the ability to infect an intermediate host, namely civet cats and camels, and then humans [34]. Detailed knowledge of how these two viruses adapted to humans could have helped us prevent COVID-19. A ‘One Health’ approach would dictate that reducing the prevalence of pathogenic viruses with a high zoonotic potential in an animal population will reduce the chance of their introduction into humans [35,36]. This is easier to accomplish with domesticated animals, where one can monitor, treat, cull, or vaccinate, but is very challenging with animals in the wild (e.g., bats). For domesticated animal populations that will naturally have extensive contact with humans, it is important to minimize conditions that promote disease such as crowding, interspecies interactions and animal stress with concomitant immunosuppression [37]. For animal populations in the wild, it is important to keep comprehensive surveillance programs to identify pathogens with known or possible transmission to humans. An example of the importance of such surveillance was provided by observations of yellow fever in howler monkeys in Brazil that foretold one of the worst outbreaks of this disease in humans in 2017–2018 [38]. Perhaps of even greater importance is the surveillance of bush meat markets, where trapped wild animals are sold as meat for food; such markets seeded the emergence of SARS-CoV-1 and SARS-CoV-2 in humans. Surveillance should include advance sequencing methods to detect the variations which could implicate enhanced zoonotic potential or pathogenicity [39]. There are many examples how these modern methods coupled with classical epidemiology result in the better understanding of infectious disease transmission, and in some cases, better outcomes [40].

Another key for the prevention of zoonoses is the protection of humans who come into contact with animals, both domesticated and wild. The first rule of protection is to prevent contact in cases where the risk is high and the need is not pressing, such as tourist visits to caves with very large bat populations. In cases where humans must be in contact with animals, risk-appropriate protocols should be in place to protect from transmission [41].

Important topics that impinge on how well we can prevent zoonotic transmission are not covered here, but include complex issues regarding climate change, agricultural practices and environmental degradation. All of these must be considered and integrated. Although there are already national (e.g., NIAID CREID network) and international groups engaged in many aspects of pandemic prevention, it is not easy to determine their effectiveness.

For arboviral diseases with pandemic potential, vector control is an important approach with a mixed track record of success. Traditional methods involve the eradication of insect vectors by manipulating the environment or by attacking the vectors directly with chemical or biological tools [42]. More recently, a promising biological intervention is to infect *Aedes aegypti* mosquitoes with the bacterium *Wolbachia pipientis* to reduce their fitness and their capacity to transmit dengue viruses, which has resulted in a significant protective effect against dengue infection [43]. Genetic engineering of vector species also promises to decrease their numbers or their vector competence for disease transmission. Noteworthy are novel gene drives coupled with methods based on RNA interference or CRISPR-Cas-based gene editing, which have been very successful in laboratory settings and promise to impact populations of vectors in the field [44,45].

Although controversial in these days of intense and sometimes irrational discussion on the origins of SARS-CoV-2, it is important to consider the intentional or unintentional adaptation of these viruses when we study them in our laboratories. There were several well-documented laboratory accidental infections with SARS-CoV-1, as there have been with many other pathogens. It is essential that all research with potentially pathogenic viruses, from the collection of potentially infected specimens from nature to the sophisticated molecular engineering of viral genomes, follows strict biosafety guidelines and protocols.

A category of ‘pandemic’ we do not consider here, but one that must be brought into the discussion, is the group of plant diseases caused by highly transmissible agents, especially RNA viruses. These have important consequences for food security and can cause significant morbidity and mortality among animal and human populations [46]. Any comprehensive response for pandemic preparedness must include voices expert in plant sciences.

For all of these prevention initiatives we must adopt global policies, since it is clear that preventing the next pandemic will not be possible unless there is strong worldwide collaboration. The social and political barriers to this are enormous, but the cost of not doing it could be catastrophic to the human race.

## 4. Managing the Next Pandemic

The SARS, MERS and COVID-19 story of the last 20 years has shown that prevention is not one of our strong suits. Furthermore, the last 36 months have shown that even in the context of a devastating pandemic, voices of reason are many times drowned by nihilism, ignorance, egotism, and stupidity. Sensible public health policies are too often sacrificed during ill-conceived political maneuvering. Therefore, it is almost a foregone conclusion that in the foreseeable future we will fail to prevent a major pandemic. We must be ready to manage it.

*Non-pharmacologic approaches.* Disease prevention measures, many considered to be tried and true, need to be based on our knowledge of the neighborhood (or suspected neighborhood if the pathogen is not unambiguously identified) of the surging pathogen. In the case of SARS-CoV-2, we should have had a trove of information on the benefits of mask-wearing based on prior experience with SARS-CoV-1 and MERS-CoV, and perhaps with other respiratory viruses, which even though genetically unrelated, can be considered neighbors because of their transmission characteristics. Why was there uncertainty in some quarters about their use? The lack of information also led to insufficient pre-pandemic investment in global production capacity and supply chains to ensure timely access of these face masks, even occasionally amongst healthcare workers looking after COVID-19 patients. Since it is far from our expertise, we do not comment on all the key social determinants of a robust public health response, but suffice it to state that our societies must evolve more systematic and effective education and a commitment to the common good.

*Vaccine anticipation.* The concept of vaccine anticipation is not new and is used yearly to prepare influenza vaccines. This approach has had a mixed record of success in reducing the burden of flu, but the concept of vaccine anticipation is reasonable and should be explored [47]. The rapid approval and administration of several effective vaccines (e.g., the Pfizer-BioNTech and Moderna COVID-19 mRNA-based vaccines, and the Oxford-AstraZeneca chimpanzee adenovirus vector vaccine) to hundreds of millions of individuals within months of isolation and the sequencing of SARS-CoV-2, is nothing short of spectacular [48,49,50]. To move even more expeditiously, we suggest that for pathogens with a high pandemic potential, we embark on developing vaccine candidates through pre-clinical efficacy studies, and clinical phase I/II clinical trials. These would show safety and immunogenicity (e.g., the appearance of neutralizing antibodies) in humans, and would be a basis to shortlist promising vaccine candidates for clinical trials as soon as the anticipated diseases emerged. This strategy, along with preapproved clinical trial protocols, could accelerate early approval and deployment by several months. RNA-based technologies, both mRNA and replicating RNA vector driven, can accelerate the timeline for the expanded manufacturing for phase 2–3 trials and clinical deployment. The establishment of the Coalition for Epidemic Preparedness Innovations (CEPI) in 2016 is a major stride in this direction, but despite their encouraging product portfolio, more can and needs to be done. Importantly, vaccine procurement and logistic pathways, along with the related financing, would all need to be agreed at an international level to ensure that those that need to be protected first have access to these vaccines equitably. This is the difference between being prepared to respond (conventional preparedness) and taking the early steps of a response before it is needed (anticipatory preparedness).

*Anticipating therapies to reduce morbidity and mortality.* The recent history with highly pathogenic human coronaviruses provides a particularly salient example of how we missed an opportunity to anticipate therapies which could have been immediately available for COVID-19. There were a few exceptional programs, such as the one that identified remdesivir as a broad-acting antiviral, but the effort should have been much broader [51]. Like all coronaviruses, SARS-CoV-1 and MERS-CoV share conserved mechanisms relating to entry, uncoating, translation of the positive-sense genome (which is also a mRNA), formation of subgenomic transcripts, genome replication, assembly, and egress [21]. Additionally, viruses in the Coronaviridae family share requirements for specific host factors, and indeed some host factors (e.g., the vacuolar ATPase) are required by viruses in many different families [52]. Viral and host factors required for efficient viral propagation represent vulnerabilities for the pathogens and thus potential targets for antivirals.

Furthermore, many viruses activate pathogenic inflammatory pathways which can be targeted to reduce morbidity and mortality; indeed, the first therapeutic to show efficacy against COVID-19 mortality was the corticosteroid, dexamethasone [53].

As an example, we should consider what could have happened if we had undertaken a comprehensive campaign to develop inhibitors of the coronavirus RNA-dependent RNA polymerases (RdRp) immediately after the SARS outbreak in 2002–2004. It is likely that several compounds significantly more potent than remdesivir could have been identified within a few years, and these could be tested for activity against RdRps from distant coronaviruses in the search of those with a broad-spectrum. Thereafter, the efficacy of promising leads in reducing SARS-CoV-1 morbidity and mortality could have been tested in relevant animal models, and the pharmacological characteristics and toxicity determined. The most promising among these antiviral candidates could have been tested in phase I clinical trials to determine tolerable doses and toxicity. As soon as MERS emerged in 2012, the efficacy of these promising antivirals could have been tested in MERS animal models and could have even been used in clinical trials with patients afflicted with MERS [18]. Optimization of these potent broad-spectrum antivirals may have provided a pre-existing weapon in the therapeutic arsenal against COVID-19. In fact, we should have embarked on comprehensive campaigns against multiple viral targets in parallel—for instance in the replicase–transcriptase complex, which includes the RdRp, other activities such as the 3′–5′ exonuclease activity of nsp14 should have been targeted. The lesson for the future is that for each of the viral families of great concern, we should identify potential targets and develop broad-spectrum potent antivirals. Whereas this would have been massively expensive, the cost of not having truly effective antivirals for the first 20 months of the COVID-19 pandemic, both in human and economic terms, was much greater.

In addition to direct viral targets, we should embark on the development of host-targeting therapies, which can be generally classified in three categories: (a) interventions that inhibit the function of a host-factor required for efficient viral replication, (b) those that enhance protective immune reactions, and (c) therapies that reduce pathologic responses such as cytokine storms. Although we argue that all three of these categories should be developed, here we use the first as an example. To identify host factors likely required by emerging pathogens, we propose that for each viral family of concern we compile an atlas of host factors and their inhibitors. Functional genome-scale methods which identify required factors using RNA interference or CRISPR-Cas gene editing/modulating loss-of-function screens have been used to identify factors exquisitely required for many viruses [54,55]. These screens can be used to identify host factors required by related viruses [56]. Exemplary studies have examined in genome-scale, the common host factor requirements for the Paramyxoviridae, mumps, measles, and Hendra viruses, and the Pneumoviridae human respiratory syncytial virus [56,57]. Some of these host factors are components of the coatomer COPI complex, which could have been predicted from our prior knowledge of the Mononegavirales order. All of these suggest that we should be assembling an atlas of host requirements, and these should be cross-referenced with catalogues of inhibitors which could be starting points for a therapeutic.

## 5. Conclusions

RNA viruses are likely to cause future pandemics and therefore we must create and organize deep knowledge of these viruses to prevent and manage this risk. Assuming prevention will fail, at least once, we must be prepared to manage a future pandemic using all resources available. We emphasize the importance of having safe vaccine candidates and safe broad-spectrum antivirals ready for rapid clinical translation. Additionally, we must have similar tools to be ready for outbreaks of RNA viruses among animals and plants. Finally, similar coordination should be accomplished for other pathogens with pandemic potential.

## Data Availability

Not applicable.
